# Digital envirotyping: quantifying environmental determinants of health and behavior

**DOI:** 10.1038/s41746-020-0245-3

**Published:** 2020-03-12

**Authors:** Matthew M. Engelhard, Jason A. Oliver, F. Joseph McClernon

**Affiliations:** Department of Psychiatry and Behavioral Sciences, Duke University School of Medicine, Durham, NC USA

**Keywords:** Risk factors, Psychiatric disorders, Cardiovascular diseases

## Abstract

Digital phenotyping efforts have used wearable devices to connect a rich array of physiologic data to health outcomes or behaviors of interest. The environmental context surrounding these phenomena has received less attention, yet is critically needed to understand their antecedents and deliver context-appropriate interventions. The coupling of improved smart eyewear with deep learning represents a technological turning point, one that calls for more comprehensive, ambitious study of environments and health.

Several major technology companies are now preparing to release consumer-friendly augmented- or mixed-reality glasses^1^, suggesting that smart, connected eyewear will soon join smartwatches on the growing list of mainstream wearable devices. These products will likely be marketed as a way to superimpose digital objects on physical ones^2^—for example, a virtual game of chess might be played on a physical tabletop—but for digital health, they represent an important new opportunity. In order to augment reality, these devices must first observe and interpret it using front-facing cameras and computer vision, respectively. In doing so, they will generate a rich visual data stream, one that complements information about user actions and physiology monitored by current consumer wearables. Unlike other digital health data streams, which focus primarily on the wearers themselves, smart eyewear will provide information about the wearer’s external world, as viewed from their perspective. Over the last decade, wearable cameras (e.g., Microsoft SenseCam) have demonstrated the value of visual data for assessing health-related behaviors and the environments in which they take place^3^. Now, building on this foundation, the coupling of next-generation eyewear with state-of-the-art computing approaches will provide a less conspicuous, more powerful means of quantifying environmental features and their impact on our health, behavior, and well-being.

The study of real-world health and behavioral status using technology has been called “digital phenotyping”, wherein data are passively and continuously captured from smartphones and connected sensors^4,5^. Through this process, wearable technologies might assess the wearer’s sleep, quantify device use and other behaviors, or even detect cardiovascular and neurological events. What often goes unmeasured, however, is the environmental context surrounding these phenomena, information that is critically needed to understand their antecedents, and to design and deliver context-appropriate interventions. Without information about context we might detect panic attacks (e.g., via heart rate and skin conductance sensors), but fail to identify the social environments that consistently trigger them. We might observe sedentary behavior and weight gain (e.g., from accelerometers and smart scales), but fail to recognize how an obesogenic work, home, or neighborhood environment discourages physical activity and healthy eating. In our own program of research, for instance, we have found that personal environments associated with smoking elicit urge and increase smoking behaviors^6^. Moreover, emerging evidence suggests that many smokers spend the majority of their time in pro-smoking environments, which may have substantial influence on their ability—or inability—to quit. When interpreting health and behavior, the environment matters.

We agree that digital health tools have a critical role to play in quantifying individual phenotypes, defined as an individual’s observable characteristics, in naturalistic settings and with greater detail and precision. Less recognized but equally compelling, however, is these tools’ imminent and expanding potential to quantify individual envirotypes, defined as the characteristics of the environments in which the individual functions, and which in turn influence their phenotype. The term envirotype, originally borrowed from the ecology literature, has previously been used to conceptualize environmental factors affecting phenotypic variability in plant growth, and in a mouse model^7^. However, the impact of the environment on health has long been recognized and named. In 1999, for example, Raudenbush and Sampson^8^ emphasized the need to go beyond aggregated administrative data when studying neighborhood characteristics, proposing the term ecometrics for the quantitative assessment of ecological settings. Subsequently, Wild^9^ introduced the exposome, defined as the complete and comprehensive history of an individual’s environmental exposures, and called for expanded, coordinated efforts to study it. In using the term envirotyping, our goal is not to add to an already crowded landscape of terms and frameworks. Rather, we aim to emphasize envirotyping as a critical complement to digital phenotyping that is facilitated by very recent and impending technological advances.

Digital phenotyping efforts have been quick to adopt new wearable devices and sensors to capture a rich array of physiologic variables. Accelerometers now track physical activity, photoplethysmography is used to estimate heart rate and pulse pressure, and skin conductance serves as a proxy for physiologic arousal^10^. Smartphone apps can monitor device use, aggregate data from multiple sources, and apply predictive models to identify behaviors of interest or stratify risk for specific health conditions^11^. Compared to physiologic and behavioral data, however, environmental factors have been more difficult to collect and analyze. GPS, the predominant source of environment-related information, is valuable for assessing overarching movement and activity patterns. However, GPS coordinates do not capture the wearer’s experience at a given location, which depends on their viewpoint as well as social and other conditions at the time. GPS might tell you that an individual is at a grocery store, but not which aisles they visited, which product displays they viewed and for how long, or who else was there, all of which are critical in understanding how retail environments affect food purchasing decisions.

Wearable cameras, on the other hand, provide direct information about the wearer’s environment, from their perspective. Devices such as Microsoft SenseCam, which was originally envisioned as a lifelogging tool, have been used to identify and contextualize physical activity^12^, and to quantify environmental correlates of eating^13^ and other behaviors. While these studies have established the research value of environmental images, they have also been limited to some degree by participants’ willingness to wear the devices^14^ as well as ethical and privacy concerns related to the intrusiveness of image collection and storage^15^. More fundamentally, prior to recent advances in computer vision, manual analysis of these images was required to identify environmental features believed to be relevant to the health or behavior of interest. Reliance on manual analysis also limited the scalability of these efforts and prevented environmental features from being analyzed or utilized in real time.

These limitations are now disappearing. Consumer-oriented devices under development by Apple^16^, Facebook^17^, and others are likely to make smart eyewear ubiquitous and mainstream, increasing participant adherence and facilitating large-scale studies. More critically, advances in deep learning (i.e., convolutional neural networks) now allow a wide range of objects and settings to be algorithmically identified, reducing or eliminating the need for manual review. With this approach, environmental features associated with symptoms or health behaviors of interest, including patient-reported outcomes, can be learned directly from observational data. In recent work, for example, we have shown that a deep learning approach can be used to identify environmental objects and settings associated with smoking^18^. Moreover, a number of deep learning models have been designed to analyze images in real time on a mobile device^19,20^, allowing image features to be immediately incorporated in a predictive model or used to trigger a just-in-time adaptive intervention^21^. Device-based image processing also allows derived features to be stored instead of storing the images themselves, reducing memory use and mitigating privacy-related concerns. Just as smartphones and consumer wearables have empowered digital phenotyping, so too will deep learning and new, smart eyewear empower a new generation of envirotyping initiatives.

As envirotyping technologies improve, a number of known, environment-related health risks should be targeted by early envirotyping initiatives. Smart eyewear might be leveraged to more precisely describe how contact with residential green space influences mental health risk^22^, or how living near fast food and tobacco retail outlets increases daily exposure to advertising, thereby increasing obesity and smoking risk, respectively. When these and many other environmental risks have been studied to date, environment characteristics have typically been aggregated by neighborhood or geographic area. This approach measures study participants’ access to salient environmental features rather than exposure itself, which may vary widely between individuals even within the same household. Studies of obesogenic environments have illustrated this limitation: self-reported and objectively measured neighborhood characteristics tend to differ, and it is the self-reported characteristics that correlate more strongly with obesity status^23^. In contrast, vision-based methods will empower future envirotyping studies by precisely, objectively quantifying exposure to environmental risk factors among individual study participants. Moreover, this strategy will allow daily environments to be characterized in unprecedented detail, thereby facilitating discovery of unknown, and perhaps unexpected, environmental determinants of health and well-being.

The coupling of smart eyewear and computer vision represent a technological turning point, one that calls for a more comprehensive, ambitious study of environments and health. For the first time, we have a practicable method to collect and analyze environmental exposures on an individual basis, yet at scale. As envirotyping technologies mature, the digital health field must prepare to take advantage of them with new, large-scale digital envirotyping initiatives, or by adding image acquisition and analysis to existing digital health initiatives such as All of Us and Project Baseline. Characterizing environmental determinants of health and behavior is an important first step toward a broader science that will include environment modification and environment-centered intervention. In labeling this process ‘digital envirotyping’, we aim to emphasize its relationship to digital phenotyping and connect geno-, pheno-, and envirotyping efforts to more comprehensively and holistically understand and improve human health.
